# Assessing the availability and impact of stress management interventions at higher education institutions: a systematic review

**DOI:** 10.3389/phrs.2026.1607815

**Published:** 2026-08-14

**Authors:** Pal Banda, Katalin Czako, Geraldine Maughan

**Affiliations:** 1 Széchenyi István University, Gyor, Hungary; 2 Technological University of the Shannon: Midlands Midwest, Limerick, Ireland

**Keywords:** anxiety management, employee wellbeing, mental health, stress management, student wellbeing

## Abstract

**Objectives:**

This systematic literature review evaluates the availability and impact of stress management interventions on mental wellbeing within higher education institutions (HEIs).

**Methods:**

A systematic review of literature was conducted across Scopus, PubMed and PsycINFO, applying the PRISMA method. Data extraction was performed manually based on pre-defined criteria. Data synthesis focuses on research design, methodology, geographical coverage, and outcomes of the interventions.

**Results:**

Thirty-two research papers met the selection criteria, exhibiting diversity in methodologies, study designs, types of interventions, outcomes, and limitations. Common patterns included a predominance of student-focused interventions and notable gaps in addressing the mental health needs of academic and administrative staff members of HEIs.

**Conclusion:**

Despite a wide range of approaches have been employed to address the mental wellbeing of students in higher education, comprehensive interventions for staff members are lacking. Additionally, there is a critical need for future research to incorporate insights and data from non-Western contexts as well, such as the CEE region, to ensure the relevance and applicability of intervention strategies across diverse cultural and socio-economic contexts.

## Introduction

Workplace stress and mental wellbeing have become increasingly important in higher education institutions (hereinafter HEIs), affecting students, administrative employees, and academic staff. High workloads, limited work-life balance, insufficient managerial support, and increasing performance expectations contribute to stress and anxiety, negatively influencing both mental health and academic or professional performance. Literature reveals that mental health disorders are strongly associated with poor academic outcomes [1]. Despite increased research on student mental health, many students do not receive adequate support [2].

Mental stress and its impact on mental wellbeing have become growing concerns in various professions [3]. Diverse approaches have been discussed to address stress and promote employees’ mental wellbeing [3–8]. Evidence suggests that employees of HEIs are substantially impacted by stress, due to elevated workloads, lack of work-life balance, insufficient support from the management, and other factors contributing to stress and anxiety, adversely affecting their mental wellbeing. This issue is becoming increasingly worrisome, representing a global challenge that affects academic staff in HEIs worldwide [9].

Certain professions, particularly those involving human contact and immediate decision-making, are more prone to elevated stress levels [5]. Among the identified psychosocial risk factors, stress, conflicts between work and family obligations, unmet emotional needs, job dissatisfaction, and burnout are the most pivotal. These factors pose substantial risks to employees’ overall mental wellbeing and might result in anxiety, insomnia, fatigue, and depression [7]. This concern is reinforced by other scholars who assert that stress is an inherent aspect of most work environments, stemming from both physical and emotional stressors, such as heavy workloads, role ambiguity, increased levels of uncertainty, and inadequate managerial support [4].

Challenges such as impaired communication, language barriers, insufficient financial resources–particularly prevalent in the non-profit sector–and the absence of work-life balance are major workplace stressors [4]. Psychological stressors include poorly defined job responsibilities, high workload, fast-paced work environment, irregular schedules, lack of control and support from the managers and colleagues [6]. Emotional exhaustion, depersonalization, and a lack of sense of accomplishment are also critical factors intensifying work-related stress [8]. Occupational stressors are categorized into work environment-related, management-related, and job demands-specific stressors. Work environment-related stressors encompass issues such as overcrowded workspaces and inadequate equipment and resources. Management-related stressors involve frequent changes in management techniques, conflicting responsibilities, multiple supervisors, discrimination and prejudice from managers, as well as the lack of recognition for achievements and emotional support from managers. Job demands-specific stressors incorporate long and inflexible working hours and the lack of time to rest [5]. The amalgamation of these workplace stressors contributes to the development of burnout [8].

Workplace health and safety regulations vary among sectors, jurisdictions, and countries. Despite the presence of these occupational health standards, employers have an integral responsibility to assist their employees in nurturing their mental health. Managers play a crucial role in establishing a supportive work environment by adopting systematic approaches. This entails a strategic and organized effort to implement measures that promote mental health within the workplace [6]. A proactive approach is needed to mitigate workplace stress, serve as a protective barrier against burnout, and recognize the capability of organizations to impact the mental health of their employees [7]. Although supervisors play a key role in managing workplace stress, employees often avoid discussing mental health concerns because of stigma and fear of inadequate support, limiting open communication [8].

Researchers have uncovered the role of trust in mental wellbeing, emphasizing the inverse correlation between workplace stress and mutual trust among colleagues and trust in management. Reduced levels of trust in management and among colleagues correlate with elevated risks of stress in the workplace [4–7]. The absence of trust in mental wellbeing support efforts undermines their effectiveness. Some discern the deficiency in open communication regarding mental health concerns within the workplace as a primary source of gaps in stress management [8]. Others scrutinize workplace stress-related risk management policies and observe deficiencies in adopting comprehensive risk management strategies, particularly in addressing non-physical factors [6]. It was found that the majority of the examined organizations primarily focus on mitigating behavioral issues, such as violence and bullying. However, a limited number of organizations incorporate strategies to address hazards such as heavy workloads and the lack of emotional support for employees [6]. The mental wellbeing of individuals within an organization is a crucial component of its strategic resilience, as the collective mental health of employees can impact the organization’s capacity to respond, recover, and thrive in the face of challenges [10].

These findings emphasize the need for more holistic approaches in organizational mental wellbeing initiatives, which underscores the rationale for this review, aiming to explore interventions that are designed to effectively manage stress and promote mental health in HEIs.

This systematic literature review aims to answer the following research questions:

Q1: Based on the existing literature, what are the prevalent gaps in the availability and impact of mental wellbeing support initiatives in HEIs targeting students, administrative staff, and academic staff members?
Q2: Which mental wellbeing interventions are considered most effective in managing stress and promoting mental wellbeing at HEIs?

For this systematic literature review, staff roles are interpreted as follows. Academic staff are primarily engaged in teaching and research activities. Teaching involves designing and delivering courses, assessing student performance, and providing academic advising. Research responsibilities include, *inter alia*, conducting research, publishing findings, and securing funding for research projects. Administrative staff members support the operational functions of HEIs. They manage essential services such as recruitment, admissions, enrollment, program coordination, student services, human resources, IT services, and maintenance.

## Methods

### Data sources

This systematic review was conducted and reported in accordance with the Preferred Reporting Items for Systematic Reviews and Meta-Analyses (PRISMA 2020) guidelines [11].

**FIGURE 1:**
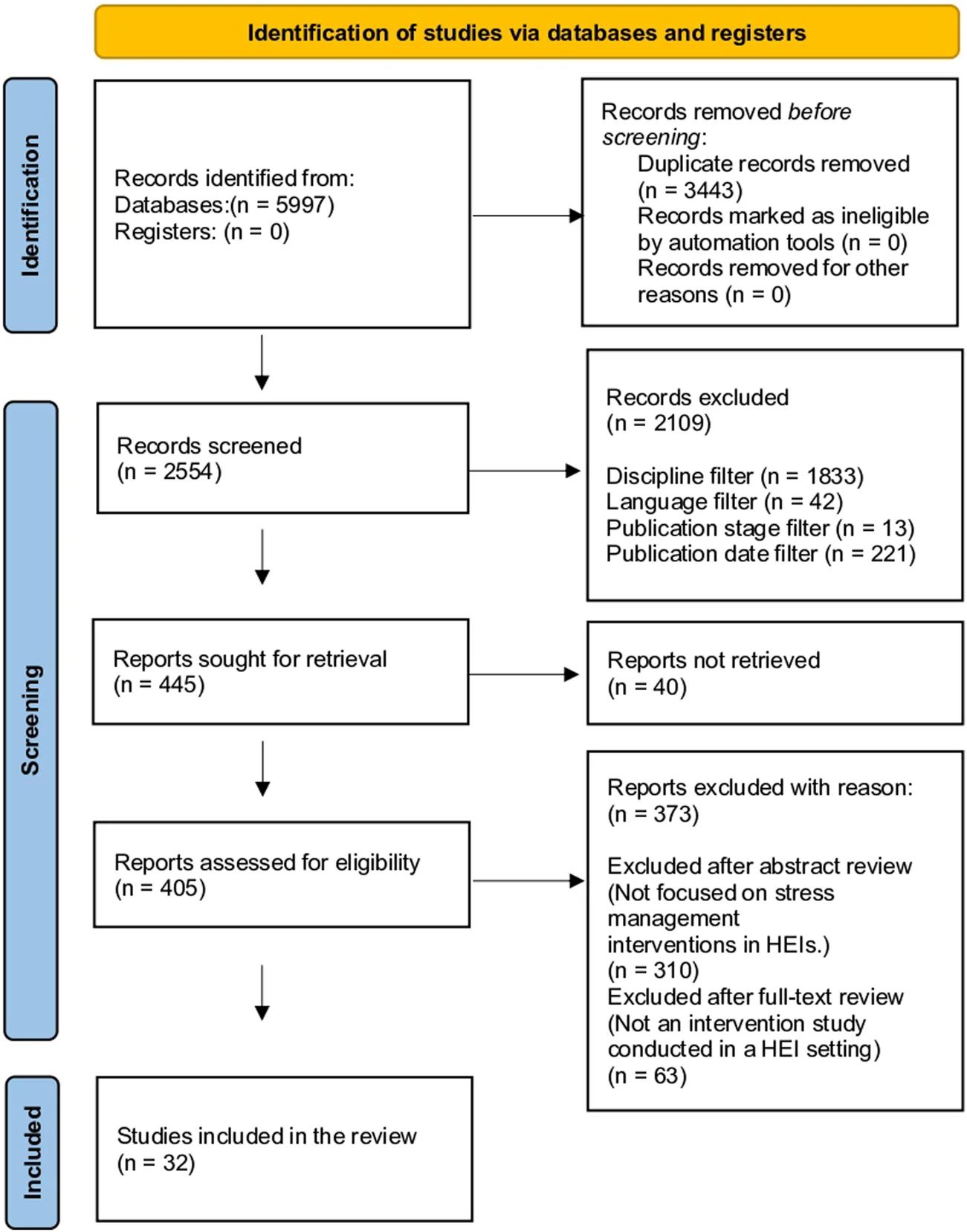
Preferred reporting items for systematic reviews and meta-analyses (PRISMA) flow chart for inclusion of studies, Hungary, 2026.

In December 2023, thorough searches of the Scopus, PubMed, and PsycINFO databases were conducted to identify pertinent literature. Study selection was conducted jointly by two authors. Titles, abstracts, and full texts were assessed against the predefined inclusion and exclusion criteria. Any uncertainties regarding study eligibility were discussed among the authors and resolved by consensus. Articles featuring a convergence of two key concepts were evaluated for suitability for inclusion. Concept 1 pertains to mental wellbeing and concept two focuses on the higher education sector. Keywords used for concept one: mental health, stress management. Keywords used for concept two: higher education, HEI, university. Each set of keywords within a concept was separated by using OR, while concepts themselves were linked by using AND. Database-specific filters were applied where appropriate. In Scopus, filters included subject area, publication year, language, and publication stage. In PubMed, publication type, publication year, and English-language filters were applied. In PsycINFO, searches were restricted to peer-reviewed journal publications and publication years 2015–2023. In total, 5997 records were identified, of which 3443 duplicates were removed, leaving 2554 records. These records were refined according to the predefined eligibility criteria. A total of 1833 studies were excluded due to discipline filters, 42 due to language filters, 13 due to publication stage filters, and 221 due to publication date filters. Forty reports could not be retrieved, leaving 405 reports for eligibility assessment. After a careful review of the title and the abstract of the located literature, manual filtering was applied to select the most relevant pieces. This rigorous process yielded a final list of 95 selected articles. The 95 identified research papers were further filtered to studies that discuss stress management interventions at HEIs. The study selection process is illustrated in Figure 1. Systematic reviews and feasibility studies were excluded because the objective of this study was to synthesize evidence from primary empirical studies evaluating stress management interventions. Besides, the review was limited to peer-reviewed publications indexed in the selected databases. Gray literature, including dissertations, conference proceedings, and institutional reports, was excluded to ensure the inclusion of studies that had undergone formal peer review and quality assessment.

### Inclusion and exclusion criteria

Inclusion criteria: 1) published between 2015 and 2023, 2) published in a final publication stage, 3) written in English, 4) discuss stress management interventions at HEIs and explore potential impediments to effective stress management practices. Exclusion criteria: 1) literature published prior to 2015, 2) literature published in languages other than English, 3) systematic reviews, 4) feasibility studies. Based on the selection according to the inclusion and exclusion criteria, 32 [12–25], [26–43] publications were chosen for this systematic literature review.

Regarding the limitation of our review to the period between 2015 and 2023, we selected this timeframe to focus on recent research to provide an up-to-date synthesis of stress management interventions within HEIs. Given the dynamic nature of mental health awareness and interventions in academic settings, focusing on the most recent years allowed us to capture the latest contributions in the field. This timeframe aligns with significant developments in mental health policy and initiatives, including the Joint Action for Mental Health and Wellbeing (JA MH-WB, representing a concerted effort involving 51 partners from 28 EU Member States and several European organizations; and the Comprehensive Mental Health Action Plan^1^ devised by the World Health Organization, both initiated in 2013. Given the launch of these initiatives in 2013 and their subsequent impact on mental health policies and practices, our decision was to commence the review from 2015 onwards. We acknowledge that this timeframe may exclude relevant studies published before 2015, and we have noted this limitation in our review.

### Data extraction

Since meta-analysis of the selected articles was not feasible due to differences in research design, such as objectives and measurement methods, this article opts for data synthesis. Data extraction was conducted by the first author and reviewed by the co-authors to ensure consistency and accuracy. Any discrepancies were resolved through discussion. Results from individual studies were tabulated and visually displayed to facilitate comparison and synthesis - detailed in Table 1. The methodological quality of included studies was evaluated by using the Risk of Bias 2.0 (RoB 2.0)^2^ tool for randomized studies–detailed in Table 2 and the ROBINS-I^3^ tool for non-randomized studies–detailed in Table 3.

**TABLE 1:** Overview of included studies, Hungary, 2026.

Authors	Year	Site country	Population	Sample size	Intervention type	Outcome measure category	Outcome assessment instrument	Assessed by (SR = self-report)	Length of the intervention	Main limitations (SR = self-report)	Research design
Abbasi S. et al	2023	Iran	Students in HE	60	Stress management training	Level of stress, anxiety, fear, depression, paranoid ideation and physical complaints	Symptom Checklist-90 (SCL90)	SR	6 weeks (one workshop per week)	Small size of sample, SR	RCT
Apolinário-Hagen J. et al	2021	Germany	Students in HE	231	Reading testimonials	Level of acceptance of digi-MHS and mediation effects	Pre-intervention: Intentions (UTAUT, pre) - SR Attitude (PU, pre) PSS Post-intervention: Source credibility, perceived similarity, intentions (UTAUT, post), attitude (PU, post), attitude, clients (APOI), attitude, public (ETAM)	SR	One semester	Multiple testing (increased risk of false positive findings and p-hacking), SR	RCT
Bendtsen M. et al	2020	Sweden	Students in HE	654	Mobile health intervention	Level of mental health, level of depression and anxiety	Mental health continuum short form (MHC-SF) Hospital anxiety depression scale (HADS)	SR	10 weeks	Lack of isolating specific themes in terms of effects, high risk of attention bias, SR	RCT
Callinan S. et al	2015	UK	Students in HE	60	Attention training (ATT)	Level of intrusion, negative affect, attention flexibility and attentional performance	Impact of Event scale (IES) - SR Positive and negative affect schedule (PANAS) Detached mindfulness questionnaire (DMQ) Attentional control capacity for emotional representations (ACCE)	IES, PANAS, DMQ: SR ACCE: Computerized performance task analyzed by researchers	2 sessions (90 and 75 min)	Lack of follow-up, SR	RCT
Chaló et al	2017	Portugal	Students in HE	50	Biofeedback	Level of anxiety and stress	Anxiety trait scale (STAI Y-2) Inventory of stress for college students (ISEU)	SR	8 weeks	Gender imbalance in sample, small size of sample size, lack of follow-up of long-term effects, SR	RCT
Chen et al	2015	China	Students in HE	71	Five-element music therapy	Level of depression	Depression mood self-report inventory for adolescence (DMSRIA) Salivary cortisol measurement (ELISA assay)	Dmsria: SR Salivary cortisol: Laboratory assay	10 weeks (2 × 40 min per week)	Convenience sample, lack of follow-up, SR	RCT
Chung et al	2021	International (Australia and UK)	Students in HE	427	Online mindfulness-based training	Wellbeing, perceived stress, mindfulness	Warwick-Edinburgh mental wellbeing scale Perceived stress scale Mindful attention awareness scale	SR	One semester	Waitlist control group, SR	RCT
Chung et al	2022	Australia	Students in HE	833	Self-managed online mindfulness program	Wellbeing, perceived stress, mindfulness	Warwick-Edinburgh mental wellbeing scale, perceived stress scale and five facet mindfulness questionnaire	SR	12 weeks	Self-selecting of participants, lack of follow-up, SR	Quasi-exerimental, pre-test — post-test design
Falsafi	2016	USA	Students in HE	90	Comparing mindfulness vs. yoga	Depressive, anxiety and stress symptoms; self-compassion; mindfulness	Beck depression inventory (BDI) Hamilton anxiety scale (HAM-A) Student-life stress inventory (SLSI) Self-compassion scale – Short form (SCS-SF) Cognitive and affective mindfulness scale–Revised (CAMS-R)	SR	8 weeks	Small sample size, gender imbalance, SR	RCT
Frazier et al	2023	USA	Students in HE	775	Web-based stress management intervention	Level of perceived stress depression, stress symptoms and anxiety and boredom	Perceived stress scale (PSS-10) Depression anxiety stress scales (DASS-21) Multidimensional state boredom scale (MSBS-15)	SR	Each intervention last 3 weeks	Self-report measures, SR	RCT
Galante J. et al	2018	UK	Students in HE	309	Mindfulness course	Level of distress, wellbeing	Clinical outcomes in routine evaluation outcome measure (CORE–OM) Warwick-Edinburgh mental wellbeing scale	SR	8 weeks	Self-report measures, only one trainer delivered the intervention, SR	RCT
Gilmore et al	2022	Australia	Students in HE	16	Telephone crisis support	Experiences and perceptions; level of distress	Semi-structured interviews	Interview responses analyzed by researchers using thematic analysis	6 months	Small sample size, gender imbalance	Interview-based qualitative study
Hashimoto et al.	2021	Japan	University lecturers	81	Suicide prevention gatekeeper training	Competence, gatekeeper behavioral intention, confidence, program satisfaction	Suicide intervention response inventory (SIRI-JS) Gatekeeper behavior questionnaire Confidence questionnaire Program effectiveness questionnaire	SR	1 h (MHL) or 2.5 h (GKT)	Lack of follow-up assessment, short interventions, non-randomized CT, SR	Quasi-exerimental, single group with non-randomized CG
Huberty et al	2019	USA	Students in HE	88	Mindfulness meditation mobile app	Perceived stress Mindfulness, self-compassion, sleep disturbance, alcohol consumption, physical activity, healthy eating	Perceived stress scale (PSS) Five facet mindfulness questionnaire (FFMQ) Self-compassion scale – Short form (SCS-SF) Patient-reported outcomes measurement information system (PROMIS) Youth risk behavior surveillance survey (YRBS)	SR	8 weeks	Gender imbalance, self-report measurement, SR	RCT
Igu et al	2023	Nigeria	University lecturers	93	Cognitive behavioral therapy with yoga	Perception of stressors and stress manifestation (level of stress, motivation, fatigue, physical conditions)	Teachers’ stress inventory (TSI) Single item stress questionnaire (SISQ)	SR	12 weeks	Small size of sample, self-report measures, waitlist control group, SR	RCT
Kourea et al	2023	International (5 countries)	Students in HE	20	BENDIT-EU program	Engagement, interest	Anonymous pilot evaluation questionnaire Open-ended feedback questionnaire	SR	5 days	Small size of sample; lack of experimental design - lack of control group	Questionnaire based pilot
Levin et al	2017	USA	Students in HE	79	Web-based acceptance and commitment therapy (ACT)	Level of stress, anxiety, depression	Counseling center assessment of psychological symptoms (CCAPS-34) Mental health continuum – Short form (MHC-SF)	SR	4 weeks	Self-report questionnaire, waitlist control groups, small sample size, SR	RCT
López-Bueno et al	2020	Spain	University staff	757	Physical activity	Level of perceived stress	Physical activity Vital sign (PAVS) questionnaire Single-item psychological wellbeing scale	SR	Not relevant	SR	Cross-sectional observational study
López-Rodríguez et al.	2017	Spain	Students in HE	121	Biodanza	Level of perceived stress and depression	Perceived stress scale (PSS) Center for Epidemiologic studies depression scale (CES-D) Pittsburgh Sleep quality index (PSQI)	SR	90 min/week - 4 weeks	Self-report questionnaire, waitlist control group, gender imbalance, SR	RCT
Lyzwinski et al	2019	Australia	Students in HE	90	Mindfulness app	Weight loss, level of stress	International physical activity questionnaire – Short form (IPAQ-SF) Three-factor Eating behavior questionnaire (TFEBQ) Perceived stress scale (PSS-10) Cognitive and affective mindfulness scale – Revised (CAMS-R) Mindful Eating questionnaire (MEQ)	SR	11 weeks	Length of the intervention, lack of no intervention CG, SR	RCT
McIndoo et al	2016	USA	Students in HE	50	Mindfulness-based therapy and behavioral activation	Level of stress, depression, ruminative response, mindfulness, treatment satisfaction	Beck depression Inventory-II (BDI-II) Hamilton rating scale for depression (HRSD) Beck anxiety inventory (Bai) Five-facet mindfulness questionnaire (FFMQ) Perceived stress scale (PSS) Ruminative response scale (RRS)	BDI-II, Bai, FFMQ, PSS, RRS: SR HRSD: Clinician-rated	4 weeks	Small sample size, SR	RCT
Morris et al	2015	UK	Students in HE	138	Internet-delivered cognitive behavior therapy (iCBT)	Level of anxiety, sleep quality, depression	State–Trait anxiety inventory – State version (STAI-S) Pittsburgh Sleep quality index (PSQI) Beck depression Inventory-II (BDI-II)	SR	6 weeks (one workshop per week)	Participant were paid, SR	RCT
Nelekar et al	2022	India	Students in HE	60	Embodied conversational agent	study stress, behaviour change intention, trust, working alliance, personality traits	Session rating scale (0–10 stress rating) Behaviour intention questionnaire Trust and Trustworthiness questionnaire Working alliance inventory – Short revised (WAI-SR) Ten item personality inventory (TIPI)	SR	12 min	Small sample size, lack of follow-up, SR	RCT
Penman et al	2019	Australia	Students in HE	89	Mental health self-management program	Mental health perceptions and practices	Pre-training questionnaire Post-training questionnaire	SR	Four two-hour sessions throughout one semester	Lack of factor analysis, SR	Quasi-experimental, pre-test — post-test design
Pignata et al	2016	Australia	University staff	869	Awareness of stress-reduction interventions	Levels of psychological strain, job satisfaction, organizational commitment	Warr job satisfaction scale (15-item) Porter organizational commitment scale (5-item) Trust in senior management scale (8-item) Procedural justice scale (4-item) Intervention awareness (IA) questionnaire item	SR	Not relevant	Lack of experimental design, SR	Cross-sectional observational study
Sousa et al	2021	Brazil	Students in HE	40	Mindfulness-based training	State mindfulness, trait mindfulness, state anxiety, trait anxiety, positive affect, negative affect, perceived stress, cortisol	State mindfulness scale (SMS) Five facets of mindfulness questionnaire (FFMQ) State-trait anxiety inventory (STAI) Positive and negative affect schedule (PANAS) Perceived stress scale (PSS-14) Plasma cortisol assay (chemiluminescence)	SMS, FFMQ, STAI, PANAS, PSS-14: SR Plasma cortisol: Laboratory assessment	90 min (30 min for 3 days)	Multiple testing with the same sample, small sample size, SR	RCT
Tay et al	2022	Singapore	Students in HE	174	Online training sessions on mental wellbeing, depression, stress	Level of depression, anxiety and wellbeing, perceived stress	Depression literacy questionnaire (D-Lit) Anxiety literacy questionnaire (A-Lit) Personal stigma scale Psychological wellbeing scale (18-item) Perceived stress scale (PSS)	SR	4 sessions (in 2 weeks)	SR	RCT
Ugwoke C. et al	2017	Nigeria	University lecturers	185	Rational-emotive health education intervention	Level of stress, irrational beliefs about teaching	Teachers’ stress questionnaire (TSQ) Teachers’ irrational beliefs questionnaire (TIBQ)	SR	10 weeks (20 sessions - each 60 min)	Small sample size, SR	RCT
Wiljer et al	2020	Canada	Students in HE	472	Mobile and web app mental health source database	Level of help-seeking intentions, help-seeking behaviors, self-stigma, self-efficacy	General help-seeking questionnaire (GHSQ) Actual help-seeking questionnaire (AHSQ) Attitudes toward seeking professional psychological help scale – Short form (ATSPPH-SF) Self-stigma of seeking help scale (SSOSH) Youth Efficacy/Empowerment scale – Mental health (YES-MH)	SR	6 months	Groups assignments were not blinded, software bugs, SR	RCT
Xu and Choi	2023	China	Students in HE	708	Participation in cultural and artistic activities	Life satisfaction	Life satisfaction scale (LSC)	SR	One time questionnaire	Lack of experimental design, sample taken from one region, SR	Cross-sectional observational study
Yang et al	2019	China	Students in HE	11954	Urban green space	Level of uncertainty and life stress	Uncertainty stress questionnaire Life stress questionnaire	SR	Not relevant	SR	Cross-sectional observational study
Yüksel and Bahadır-Yılmaz	2019	Turkey	Students in HE	91	Peer mentoring program	Level of adjustment, level of coping with stress	Adjustment to university scale (AUS) Ways of coping inventory (WCI)	SR	8 weeks	Non-randomized control group, SR	Quasi-exerimental, pre-test — post-test design (non randomized)

**TABLE 2:** Detailed risk of Bias 2.0 (RoB 2.0) assessment of randomized controlled trials, Hungary, 2026.

Study	D.1 randomization process	D.2 deviations from intended interventions	D.3 missing outcome data	D.4 measurement of outcome	D.5 selection of reported result	Overall risk of bias
Abbasi [12]	Low	High	Low	Low	Low	High
Frazier et al [21]	Low	Low	Low	Low	Low	Low
Igu et al [26]	Low	Low	Low	Low	Low	Low
Tay et al [38]	High	Low	Low	Low	Low	High
Apolinario-Hagen [13]	Low	Low	Low	Low	High	High
Chung [19]	High	High	Low	High	Some concerns	High
Nelekar [35]	High	Low	Low	High	Low	High
Sousa [37]	Low	Low	Low	Low	Low	Low
Bendsten [14]	Low	Some concerns	Low	Low	Low	Some concerns
Wijer et al [40]	Low	Some concerns	Low	Low	Low	Some concerns
Huberty et al. [25]	Low	Low	Low	Some concerns	Low	Some concerns
Lyzwinski et al [31]	High	Low	Low	Low	Low	High
Galante et al [22]	Low	Low	Low	Low	Low	Low
Chalo [16]	Low	Low	Low	Low	Low	Low
Levin [28]	Low	Low	Low	Low	Some concerns	Some concerns
López-Rodríguez et al [30]	Low	Low	Low	Low	Low	Low
Ugwoke et al [39]	Low	Low	Low	Low	Low	Low
Falsafi [20]	High	Low	​	High	Low	Some concerns
McIndoo [32]	Low	Low	Low	Low	Low	Low
Callinan [15]	Some concerns	High	Low	Low	Low	High
Chen [17]	Low	Low	Low	Low	Low	Low
Morris [33]	Low	Low	Low	Low	Low	Low

**TABLE 3:** Detailed risk of Bias in non-randomised studies of interventions (ROBINS-I) assessment of non-randomized studies, Hungary, 2026.

Study	D.1 confounding	D.2 selection of participants	D.3 classification of interventions	D.4 deviations from intended interventions	D.5 missing data	D.6 measurement of outcomes	D.7 selection of reported result	Overall risk of bias
Xu and Choi [41]	Serious	Moderate	Moderate	Moderate	Low	Serious	Moderate	Serious
Kourea et al. [27]	Serious	Moderate	Low	Moderate	Moderate	Serious	Moderate	Serious
Chung et al. [18]	Serious	Moderate	Low	Moderate	Serious	Moderate	Moderate	Serious
Gilmore et al. [23]	Moderate	Moderate	Low	Low	Low	Moderate	Moderate	Moderate
Hashimoto et al. [24]	Serious	Moderate	Low	Low	Low	Moderate	Moderate	Serious
López-Bueno et al. [29]	Serious	Moderate	Low	Low	Serious	Moderate	Moderate	Serious
Penman et al. [34]	Serious	Moderate	Low	Low	Low	Moderate	Moderate	Serious
Yang et al. [42]	Moderate	Moderate	Low	Low	Low	Moderate	Moderate	Moderate
Yüksel and Bahadır-Yılmaz [43]	Moderate	Moderate	Low	Low	Low	Moderate	Moderate	Moderate
Pignata et al. [36]	Serious	Moderate	Low	Low	Low	Moderate	Moderate	Serious

### Data synthesis

The data synthesis focuses on the primary and secondary outcomes of the identified studies. Data synthesis focused on study population, sample size, main objectives, type and length of interventions, outcome measures, effectiveness, and methodological rigor.

Data synthesis was conducted by the first author after joint consultation sessions with the corresponding authors. The consultations aimed at ensuring consensus on the synthesis process.

To synthesize the impact of the findings, a narrative approach was adopted. The choice of a narrative synthesis was justified by the need to accommodate diverse study designs and outcome measures, which precluded a quantitative meta-analysis.

## Results

### Overview of included studies

The selection of articles comprised 31 quantitative studies [12–22], [24–43] and one qualitative study [23], utilizing various study designs. Predominantly, the designs consisted of randomized controlled trials [12–17], [19–22], [25, 26, 28], [30–33], [35], [37–40], followed by quasi-experimental pre-post assessments [18, 24, 34, 43], and cross-sectional studies [29, 36, 41, 42]. Additionally, the review encompasses one interview-based qualitative study [23] and one questionnaire-based pilot study [27], as indicated.

### Main findings

This section summarizes the methodological characteristics, strengths, and limitations of the included studies and provides context for interpreting the findings.

Study population: The study population comprises students and academic staff in HEIs across various countries, including China [17, 41, 42], the US [20, 21, 25, 28, 32], Nigeria [26, 39], Iran [12], Australia [19, 23, 31, 34, 36], India [35], Brazil [37], Germany [13], Japan [24], Spain [29, 30], Turkey [43], Portugal [16], and the UK. [15, 22, 33]. Additionally, two studies feature an international sample [18, 27]. Among the included studies, the primary focus is on students’ mental health [12–23], [25, 27, 28]^,^ [30–35], [37, 38, 40–43], with a limited number examining the experiences and perspectives of university staff and lecturers [24, 26, 29, 36, 39]. Sample sizes vary across different groups, ranging from 16 [23] to 11954 [42]. The studies were published across a span of eight years, with the most recent publications from 2023, indicating a sustained interest in the subject matter over time.

The main aim of the studies: The primary objective of the included studies was to evaluate the effectiveness of interventions designed to reduce stress, enhance coping, and promote mental wellbeing among students and staff in HEIs. Through various methodologies and approaches, the studies sought to explore the efficacy of interventions aimed at alleviating stress symptoms, enhancing coping mechanisms, and ultimately fostering a positive psychological state among participants.

The nature of the interventions: Interventions in the reviewed studies are categorized into five groups, each targeting stress management and mental wellbeing through distinct approaches, as outlined in Table 4. These categories encompass traditional therapeutic interventions [12, 24, 39], technology-based interventions [14, 18, 21, 23, 25, 28, 31, 33, 35, 40], lifestyle-based interventions [26, 29, 41, 42], social and community-based interventions [36, 43], and alternative and complementary approaches [13, 15–17, 19, 20, 22, 27], [30–32, 34, 37, 38]. Each category offers unique strategies to address stress and promote mental health across various populations and settings.

**TABLE 4:** Categories of interventions, Hungary, 2026.

Intervention category	Intervention
Therapeutic interventions	Stress management training [12]
​	Suicide prevention gatekeeper training [24]
​	Rational-emotive health education intervention [39]
Technology-based interventions	Self-managed online mindfulness programs [18]
​	Web-based stress management programs [21]
​	Telephone crisis support [23]
​	Web-based acceptance and commitment therapy [28]
​	Internet-delivered cognitive behavior therapy [33]
​	Embodied conversational agents [35]
​	Mobile health intervention [14, 25, 31, 40]
Lifestyle-based interventions	Physical activity programs [26, 29]
​	Participation in cultural and artistic activities [41]
​	Exposure to urban green spaces [42]
Social and community interventions	Awareness-raising initiatives [36]
​	Peer support programs [43]
Alternative and complementary approaches	Reading testimonials on digital stress management training [13]
​	Attention training [15]
​	Biofeedback [16]
​	Music therapy [17]
​	Comparative studies of mindfulness vs. Yoga [20]
​	Burnout education, burnout management [27]
​	Biodanza [30]
​	Behavioral activation [32]
​	Mindfulness-based therapy [19, 22, 31, 37, 38]
​	Mental health self-management program [34]

The length of the interventions: The duration of interventions varied considerably across the reviewed studies. Interventions ranged from brief one-time questionnaires [41] to more extended programs spanning several months. Some interventions were conducted over short periods, such as a couple of minutes [15, 24, 35], days [27, 37], while others over several weeks [12, 14, 16–18, 20–22, 25], [26, 28, 30–33], [38, 39, 42, 43], or an entire academic semester [13, 19, 23, 34, 40]. Additionally, intervention lengths varied based on the format, with some consisting of weekly sessions over several weeks, while others were condensed into intensive workshops or single sessions.

Outcome measures: Table 5 offers a comprehensive overview of the outcome measures employed throughout the studies under review, encompassing a wide range of categories. These categories include psychological indicators, engagement metrics, mental wellbeing assessments, trust and alliance evaluations, acceptance and competence assessments, university adjustment and coping measures, and various other psychological measures.

**TABLE 5:** Outcome measures, Hungary, 2026.

Outcome measure category	Examples
Psychological indicators [12, 14, 16, 17], [20–23, 25, 26], [28–33], [35, 37–39, 41, 42]	Life satisfaction, (perceived) stress levels, depression, anxiety, boredom, fear, paranoid ideation
Engagement [18, 23, 27]	Engagement, interest, experiences, perceptions
Mental wellbeing assessment [14, 18, 19]	Level of mental health, warwick-Edinburgh mental wellbeing scale, perceived stress scale, five facet mindfulness questionnaire
Trust and alliance [35]	Trustworthiness, working alliance
Acceptance and competence [13, 24]	Acceptance of wellbeing intervention, competence in managing students with mental issues
University adjustment and coping [43]	Adjustment to university (AUS scale), coping with stress (ways of coping inventory)
Other psychological measures [15, 25, 32, 36, 40]	Uncertainty stress, life stress, psychological strain, job satisfaction, organizational commitment, ruminative response, self-compassion, intrusion, negative affect, attention flexibility, attentional performance, level of health seeking intention

A detailed overview of the outcome assessment instruments and assessors of the included studies is presented in Table 1. Most studies assessed outcomes using validated self-report surveys. Frequently used instruments included the Perceived Stress Scale, Warwick-Edinburgh Mental Wellbeing Scale, Depression Anxiety Stress Scale, General Help-Seeking Questionnaire, and Five Facet Mindfulness Questionnaire. Only a limited number of studies supplemented self-reported outcomes with other, non-self-reported measures. These include salivary cortisol assessment, plasma cortisol analysis, computerized performance tasks, laboratory-based physiological measures, or qualitative interview analysis.

### Assessing the methodological quality of the studies included

While the selected studies employ varying research designs–explored in Table 1, including randomized controlled trials [12–17, 19–22, 25, 26, 28], [30–33], [35], [37–40] quasi-experimental pre-post assessments [18, 24, 34, 43], cross-sectional studies [29, 36, 41, 42], one questionnaire-based pilot study [27], and one interview-based qualitative study [23], there are notable differences in methodological rigor across the literature. Some studies lack experimental designs [27, 36, 41], relying instead on one-time questionnaires or surveys, which may limit the ability to establish causal relationships or assess long-term intervention effects. Additionally, the use of self-report measures [17, 18, 20–22, 25, 26], [28–30, 38, 39, 42] without validation through factor analysis or physiological measures may introduce reporting biases and limit the robustness of findings. Moreover, several studies exhibit unreasonably small sample sizes [16, 23, 27, 32, 35, 37], which may reduce the generalizability and statistical power of results. Furthermore, the absence of control groups [19, 23, 24, 27, 29, 34, 36, 41, 42] or the use of waitlist controls [18, 26, 28, 30, 31] in some studies may introduce potential biases in the assessment of intervention effects. Gender imbalances in samples are also noted in more studies [16, 20, 23, 25, 30], which could impact the validity of the results. Furthermore, the absence of follow-up assessments in many studies prevented the evaluation of sustained intervention effects.

Summarizing the quality of study design across the included literature reveals variability in methodological rigor. Studies employing rigorous designs such as randomized controlled trials were deemed to have higher methodological quality due to their ability to establish causality and minimize bias. Quasi-experimental pre-post assessments and cross-sectional studies were also considered, albeit with some limitations in terms of causal inference and generalizability. Studies with larger sample sizes were considered to have higher methodological quality, as they are more likely to detect significant effects and provide more representative insights into the target population. Similarly, studies incorporating control groups were perceived to have higher methodological quality, as they allow for comparisons between intervention and control conditions, thereby strengthening causal inference. Furthermore, studies conducting follow-up assessments were regarded as having higher methodological quality, as they provide insights into the durability of intervention effects beyond the immediate post-intervention period.

### Assessing the risk of bias of included studies

To assess the risk of bias, randomized controlled trials were assessed using the Risk of Bias 2.0 (RoB 2.0) tool, developed by Cochrane. The RoB 2.0 is designed to evaluate the risk of bias of randomized studies in 5 domains, including the randomization process, deviations from intended interventions, missing outcome data, measurement of the outcome, and selection of the reported results. In each domain several questions must be answered by selecting one of the five potential answers: yes, probably yes, no, probably no and not enough information. Overall, each domain is evaluated as low risk of bias, some concerns or high risk of bias. Based on the evaluation of the five domains, a final judgement of overall risk of bias is calculated by the algorithm.

For non-randomized studies the risk of bias was assessed by using the Risk of Bias in Non-Randomized Studies of Interventions (ROBINS-I) tool, developed by Cochrane. This tool evaluates the risk of bias in 7 domains, including bias due to confounding, bias due to selection, bias due to the classification of the intervention, bias due to deviations from the intended intervention, bias due to missing data, bias in outcome measurement, and bias in the selection of reported results. Each of these domains is assessed as low risk, moderate risk, serious risk or critical risk of bias.

The risk-of-bias assessment was initially conducted by the first author using the RoB 2.0 and ROBINS-I tools. Assessments that were considered uncertain or difficult to classify were subsequently reviewed and discussed with a co-author, and final judgments were reached through consensus. Out of the twenty-two randomized controlled trials evaluated, ten [16, 17, 21, 22, 26, 30, 32, 33, 37, 39] were found to have a low risk of bias. Five studies had some concerns regarding bias [14, 20, 25, 28, 40], while seven [12, 13, 15, 19, 31, 35, 38] were deemed to have a high risk of bias, necessitating cautious interpretation of their findings. Out of the ten non-randomized studies evaluated, seven [18, 24, 27, 29, 34, 36, 41] were found to have a serious risk of bias, which may affect confidence in their findings. Three studies [23, 42, 43] were assessed as having a moderate risk of bias, suggesting some concerns in the devised methodology. An overview of the risk of bias assessments can be found in Table 1.

### Assessing the effectiveness and impact of interventions

Interventions targeting stress and mental wellbeing at HEIs have been subject to rigorous evaluation through randomized controlled trials and empirical studies. Xu and Choi [41] uncovered a significant positive correlation between engagement in cultural and artistic activities and heightened life satisfaction among university students, highlighting unconventional avenues for bolstering psychological resilience. Frazier et al [21] demonstrated the efficacy of web-based stress management interventions during the COVID-19 pandemic, underscoring the pivotal role of accessible online platforms in alleviating stress symptoms among college students. Similarly, Igu et al [26] emphasized the value of holistic approaches by integrating cognitive behavioral therapy (CBT) with yoga to reduce job stress among university lecturers, showcasing promising strategies for addressing occupational stressors within academic environments. Abbasi et al [12] investigated stress management training for medical students, which yielded notable reductions in both physical complaints and psychological distress, reaffirming the importance of targeted interventions in fostering mental wellbeing within educational contexts. Complementing these findings, Gilmore et al [23] considered the challenges faced by participants in a telephone crisis support training program, emphasizing the necessity of comprehensive support mechanisms for those involved in crisis intervention roles. These studies, alongside others such as Hashimoto et al [24] and Sousa et al, [37] collectively highlight the diverse yet interconnected approaches and positive outcomes of interventions, underscoring the ongoing imperative for research and implementation efforts in promoting mental health within university communities.

Overall, mindfulness-based interventions and CBT-based approaches demonstrated the most consistent positive outcomes across the reviewed studies. Interventions supported by randomized controlled trials and lower risk of bias assessments provided stronger evidence of effectiveness in reducing stress, anxiety, and depression. In contrast, interventions assessed through cross-sectional or non-randomized designs provided less conclusive evidence regarding effectiveness. These findings suggest that mindfulness-oriented and CBT-based interventions currently represent the most promising approaches for promoting mental wellbeing in HEIs, although further longitudinal research is required to confirm sustained effectiveness.

A notable observation emerging from the assessment of intervention impact is the prevalent focus on students within HEIs, with a lack of interventions tailored specifically for academic and administrative staff. The available evidence also suggests differences in the focus of interventions across population groups. Student-targeted interventions primarily addressed individual psychological outcomes, including stress, anxiety, depression, mindfulness, wellbeing, resilience, and help-seeking behaviours. In contrast, the smaller number of studies involving university staff tended to focus on occupational outcomes such as job stress, psychological strain, burnout, job satisfaction, organizational commitment, and workplace coping. These findings suggest that students and staff may experience different stressors and support needs, highlighting the importance of developing and evaluating interventions tailored to the specific challenges faced by each group. Given that staff wellbeing is equally important to the overall functioning of HEIs, future research should prioritize the development and evaluation of interventions for academic and administrative staff to support a more comprehensive and inclusive approach to mental health promotion within higher education settings.

Another observation worth mentioning is the limited geographical diversity in the locations where the interventions were conducted and evaluated. While the selected studies encompass a range of countries, most of the interventions are still predominantly concentrated in Western contexts, with no representation from regions such as Central and Eastern Europe. This geographical bias raises questions about the generalizability of findings and the applicability of intervention strategies across diverse cultural and socio-economic contexts. The lack of representation from regions with distinct educational systems, cultural norms, and socioeconomic conditions underscores the need for more inclusive and culturally sensitive approaches to intervention development and evaluation. Future research should strive to incorporate a more diverse range of settings and populations, including those from Central and Eastern Europe, to ensure that interventions are effective and relevant across different contexts, ultimately contributing to more equitable and inclusive mental health support within higher education globally.

## Discussion

### Summary of the main findings

This review synthesized evidence on stress management interventions implemented within HEIs. The findings suggest that a wide range of intervention approaches have been applied, including mindfulness-based programmes, cognitive-behavioural interventions, psychoeducational approaches, and skills-development programmes. Overall, the reviewed studies suggest that such interventions may contribute to improved stress management and mental wellbeing among participants. However, considerable heterogeneity was observed across intervention types, study designs, intervention duration, outcome measures, and sample characteristics, limiting direct comparisons across studies. Predominantly, studies utilized randomized controlled trials. Intervention lengths varied widely, from brief single-session programmes to extended interventions. Sample sizes also varied substantially, with some studies including relatively small samples that may limit generalizability and statistical power. Furthermore, many studies relied on self-report measures, which may affect the robustness of findings. Most studies relied exclusively on self-reported outcomes, whereas only a small number of incorporated non-self-reported measures. Among these studies, three were rated as having a low overall risk of bias, one as moderate risk, and one as high risk. Although this may suggest a tendency toward stronger methodological rigor among some studies employing more diverse assessment approaches, the small number of such studies does not allow for firm conclusions.

The findings of the present review are broadly consistent with previous systematic reviews conducted in higher education settings. Regehr et al. reported that cognitive, behavioral, and mindfulness-based interventions were effective in reducing student stress [44]. Amanvermez et al. found moderate effects of stress management interventions on stress, depression, and anxiety among college students [45]. Similarly, Worsley et al. highlighted the positive effects of mindfulness- and cognitive behavioural-based approaches on student mental health and wellbeing [46]. The consistency of findings across previous reviews and the present review suggests that mindfulness-based and cognitive-behavioral approaches represent the most promising evidence-based strategies currently available for stress management within higher education settings.

This review identified a notable lack of studies targeting academic and administrative staff. This highlights an important gap in the current evidence base and suggests that future intervention research should extend beyond student populations. The available evidence also suggests differences in the focus of interventions across population groups. Student-targeted interventions primarily addressed individual psychological outcomes, including stress, anxiety, depression, mindfulness, wellbeing, resilience, and help-seeking behaviours. In contrast, the smaller number of studies involving university staff tended to focus on occupational outcomes such as job stress, psychological strain, burnout, job satisfaction, organizational commitment, and workplace coping. These findings indicate that students and staff experience different stressors and support needs, highlighting the importance of developing and evaluating interventions tailored to the specific challenges faced by each group.

### Limitations

This systematic literature review has limitations. The selected databases may not have captured all relevant literature and primarily index peer-reviewed publications; consequently, grey literature (e.g., conference proceedings, dissertations, unpublished studies, and institutional reports) was excluded, potentially increasing publication bias. In addition, only English-language studies were included, and the keyword-based search strategy may have overlooked relevant studies using alternative terminology. Besides, the risk-of-bias assessments were initially conducted by one author, although uncertain cases were reviewed and discussed with the co-authors to reach consensus, the initial assessment by a single author, may have increased the risk of subjective judgment. Furthermore, substantial heterogeneity was observed across intervention types, study designs, outcome measures, outcome assessment instruments, outcome assessors, and reporting practices. Information regarding intervention delivery, participant adherence, intervention fidelity, and assessment time points was often inconsistently reported across the included studies. As a result, direct comparisons across studies were limited, and a quantitative meta-analysis was not feasible. The findings of this review are based on a narrative synthesis of the reported outcomes.

## Conclusion

This review synthesized evidence on stress management and mental wellbeing interventions implemented within HEIs. The findings suggest that a variety of intervention approaches, including mindfulness-based, cognitive-behavioural, psychoeducational, and skills-development programmes, may contribute to improved stress management and mental wellbeing. However, substantial heterogeneity across interventions, study designs, and outcome measures, assessment instruments and assessors limits direct comparisons of effectiveness. The review highlights a predominant focus on student-tailored interventions, with limited representation of interventions designed specifically for academic and administrative staff. Expanding the evidence base beyond student-focused interventions will be essential for developing a more comprehensive understanding of how stress management strategies can support the wellbeing of all stakeholders within higher education institutions. Additionally, the lack of studies from diverse regions, such as from the Central and Eastern European region, underscores a notable gap in the existing literature, pointing to the importance of incorporating heterogeneous settings and populations to ensure the generalizability and applicability of intervention strategies across different cultural and socio-economic contexts. Future research may prioritize longitudinal studies, delve into participant outcomes in greater depth, and critically assess the limitations of existing interventions.

## Footnotes

1 WHO. Comprehensive Mental Health Action Plan 2013–2030. www.who.int. Published September 21, 2021. https://www.who.int/publications/i/item/9789240031029

2 Cochrane. Risk of Bias 2 (RoB 2) tool. methods.cochrane.org. Published 2017. https://methods.cochrane.org/risk-bias-2

3 Cochrane. ROBINS-I tool. methods.cochrane.org. Published 2016. https://methods.cochrane.org/robins-i
